# Integrated hepatitis B virus DNA maintains surface antigen production during antiviral treatment

**DOI:** 10.1172/JCI161818

**Published:** 2022-09-15

**Authors:** Tanner Grudda, Hyon S. Hwang, Maraake Taddese, Jeffrey Quinn, Mark S. Sulkowski, Richard K. Sterling, Ashwin Balagopal, Chloe L. Thio

**Affiliations:** 1 Department of Molecular Microbiology and Immunology, Johns Hopkins Bloomberg School of Public Health, Baltimore, Maryland, USA.; 2 Department of Medicine, Johns Hopkins University School of Medicine, Baltimore, Maryland, USA.; 3 Division of Gastroenterology, Hepatology, and Nutrition, Virginia Commonwealth University, Richmond, Virginia, USA.

**Keywords:** Infectious disease, Virology, Antigen, Hepatitis, Molecular biology

## Abstract

The focus of hepatitis B functional cure, defined as sustained loss of hepatitis B virus (HBV) surface antigen (HBsAg) and HBV DNA from blood, is on eliminating or silencing the intranuclear template for HBV replication, covalently closed circular DNA (cccDNA). However, HBsAg also derives from HBV DNA integrated into the host genome (iDNA). Little is known about the contribution of iDNA to circulating HBsAg with current therapeutics. We applied a multiplex droplet digital PCR assay to demonstrate that iDNA is responsible for maintaining HBsAg quantities in some individuals. Using paired bulk liver tissue from 16 HIV/HBV-coinfected persons on nucleos(t)ide analog (NUC) therapy, we demonstrate that people with larger HBsAg declines between biopsies derive HBsAg from cccDNA, whereas people with stable HBsAg levels derive predominantly from iDNA. We applied our assay to individual hepatocytes in paired tissues from 3 people and demonstrated that the individual with significant HBsAg decline had a commensurate loss of infected cells with transcriptionally active cccDNA, while individuals without HBsAg decline had stable or increasing numbers of cells producing HBsAg from iDNA. We demonstrate that while NUC therapy may be effective at controlling cccDNA replication and transcription, innovative treatments are required to address iDNA transcription that sustains HBsAg production.

## Graphical abstract

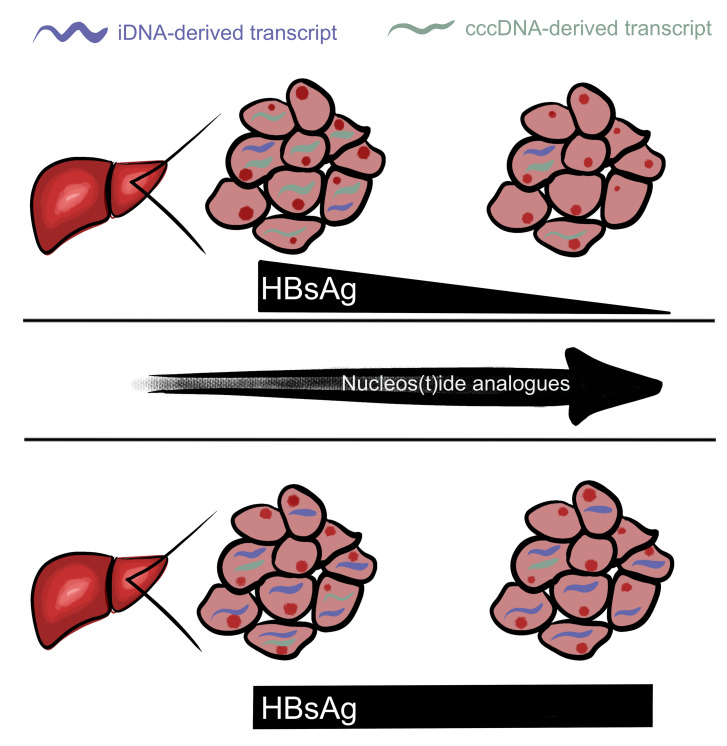

## Introduction

For the nearly 300 million people chronically infected with hepatitis B virus (HBV), current treatments, which include pegylated interferon α (PEG-IFN-α) and nucleos(t)ide analogs (NUCs), uncommonly result in cure, defined as a sustained loss of detectable HBV surface antigen (HBsAg) and HBV DNA in blood after cessation of therapy (1–5). NUCs inhibit HBV replication by interrupting HBV polymerase, a reverse transcriptase that converts viral pregenomic RNA (pgRNA) to relaxed circular DNA (rcDNA), the infectious genome detected in plasma. Importantly, NUCs do not directly affect the covalently closed circular DNA (cccDNA) present in the nucleus of every infected cell, and thus cannot, on their own, result in HBV eradication (6–8). Furthermore, although NUCs are the mainstay of antiviral treatment in chronic HBV (CHB), their suppressive effect on HBV viremia belies their limited impact on plasma HBsAg levels (1, 9, 10).

Although a central tenet of functional HBV cure is sustained loss of HBsAg in blood, it is important to acknowledge that HBsAg is a heterogeneous group of related proteins; distinct surface (*S*) mRNAs correspond to 2.4 kb (large ORF) and 2.1 kb (middle and small ORFs) transcripts. In addition, *S* mRNAs can be transcribed from either cccDNA or numerous integrated HBV DNAs (iDNA) found interspersed throughout the human genome (8, 11–13). iDNAs are stable integrands of the truncated viral genome capable of producing complete *S* ORF transcripts in abundance. They can be distinguished from cccDNA-derived transcripts because iDNA-derived *S* mRNAs are chimeric with host DNA and lack the common 3′ ends that all cccDNA-derived transcripts share (8, 11–13). Previously, we used single-cell laser capture microdissection (scLCM) to report that in persons taking long-standing NUCs, cccDNA transcription was markedly diminished and even silenced (14, 15). While intriguing on its own, the observation raises the question as to why HBsAg levels did not decline commensurately during NUC treatment. We hypothesized that the continued production of HBsAg in persons taking NUCs is due to continued *S* mRNA transcription from iDNA.

To address this hypothesis, we developed a multiplex droplet digital PCR (ddPCR) assay that distinguishes between cccDNA- and iDNA-derived *S* transcripts. We applied our assay to paired bulk liver biopsies from individuals in a well-characterized HIV/HBV coinfection cohort who received NUCs and had longitudinal follow-up. We linked hepatic measurements with peripheral quantities of HBsAg. In a subset of these paired biopsies, we combined scLCM and multiplex ddPCR to compare levels of cccDNA-derived and iDNA-derived *S* transcripts in individual hepatocytes.

## Results

### A multiplex ddPCR assay for iDNA-derived transcripts.

**Figure 1:**
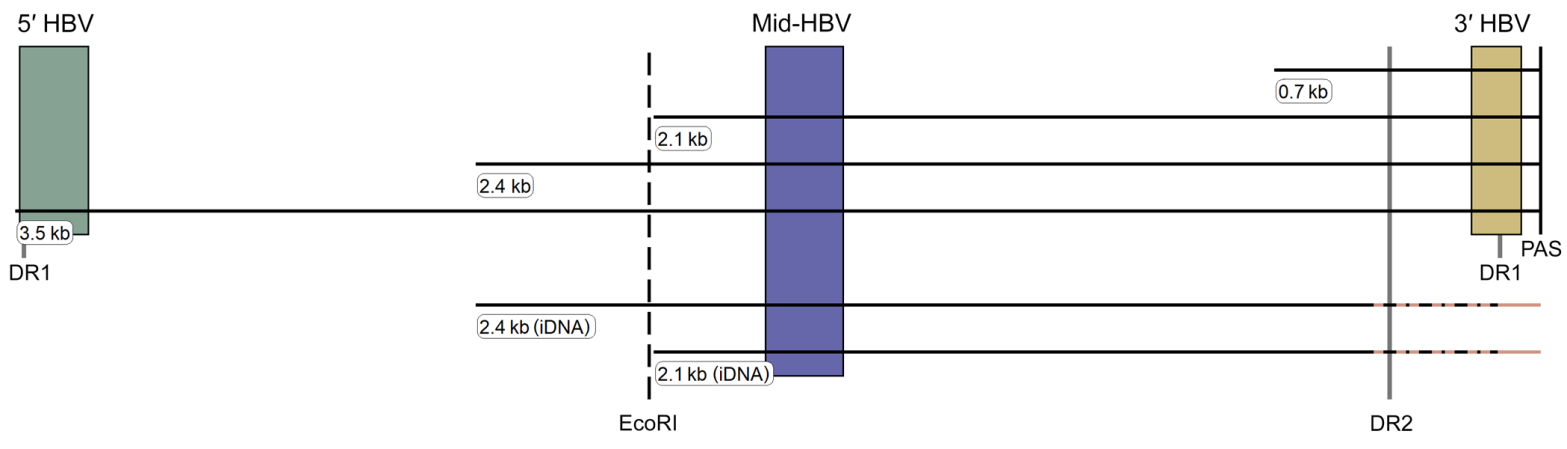
HBV transcriptional map and multiplex ddPCR assay targets. Horizontal lines depict HBV transcripts derived from cccDNA or iDNA (labeled). The variable chimeric virus-host region is shown as hashed lines at the 3′ end of iDNA-derived transcripts. The dashed vertical line is the EcoRI cut site for orientation. Solid vertical lines show the locations of the DR1, DR2, and poly-A signal (PAS) regions for reference. Individual targeted assays for each component of the multiplex ddPCR are shown as vertical boxes spanning the shaded regions at the 5′, middle, and 3′ ends of HBV transcripts.

We developed a discriminatory assay exploiting the shared cccDNA-derived mRNA 3′ overlaps that terminate in a common poly-A signal (PAS). Previously published long- and short-read sequencing transcriptome maps of HBV-infected liver tissues indicate that iDNA-derived transcripts, overwhelmingly *S* mRNAs, lack the common PAS (8, 11–13). Thus, we designed a multiplex ddPCR assay to independently quantify the middle (mid-HBV) and 3′ (3′ HBV) ends of HBV mRNAs to distinguish transcripts that derive from cccDNA from those that derive from iDNA. The mid-HBV assay was designed to align against all *S* mRNAs as well as 3.5 kb mRNA if present. We added a 5′ assay (5′ HBV) that quantifies pgRNA as an independent measure of transcriptional activity from cccDNA since transcriptome maps rarely demonstrate iDNA-derived pgRNA (Figure 1). Using these multiple ddPCR assays in combination, iDNA-derived transcripts can be distinguished from cccDNA-derived transcripts when the mid-HBV region is amplified but the 3′ HBV assay is not. We verified the specificity of this approach by applying these assays to transcripts taken from Hep3B cells, which only contain iDNA, that were added to HBV-uninfected liver tissue and diluted. We confirmed that only the mid-HBV amplicon, and not the 3′ HBV amplicon, was quantifiable over a dynamic range (Supplemental Figure 1; supplemental material available online with this article; https://doi.org/10.1172/JCI161818DS1). Thus, we concluded this approach was specific for iDNA-derived transcripts. We applied the same multiplex assay to liver from a mouse model of HBV infection (see Methods). C57BL/6 mice were transfected with a cccDNA expression vector from which all canonical HBV transcripts are produced. We applied the mid-HBV and 3′ HBV assays to homogenized liver extracts from these mice and found that both assays quantified abundant transcripts over a dynamic range, confirming their sensitivity (Supplemental Figure 2). In addition, we tested each assay individually and in combination against purified synthetic DNA standards in equimolar concentrations across a range of dilutions and found comparable PCR efficiencies (Supplemental Figure 3). The lower limit of detection for each amplicon was less than 10 copies/μL input. Finally, we verified in silico that these primers would work across genotypes by demonstrating that our primers and probes bind equally to more than 4,000 full-length HBV sequences of varying genotypes (Supplemental Table 3).

### Multiplex ddPCR assay correlates with HBsAg levels in blood.

**Figure 2:**
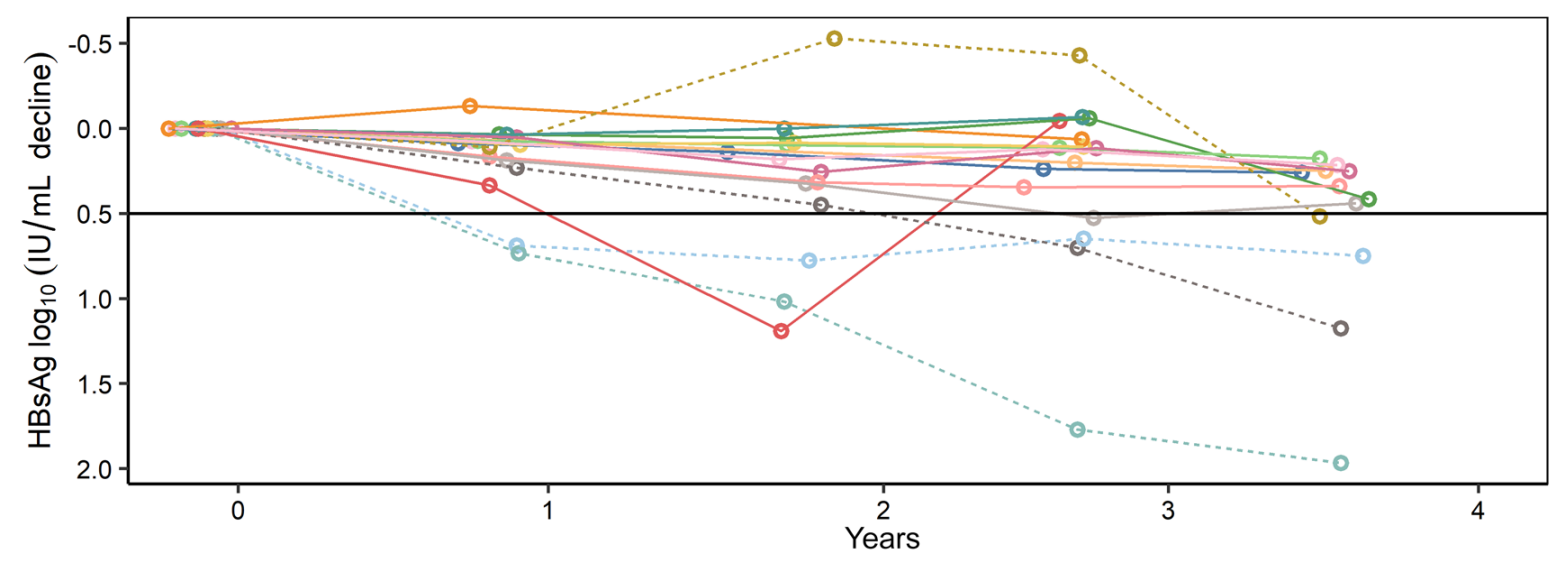
Serum HBsAg over time. Serum HBsAg declines are shown for each person between the time of their liver biopsies. Each person (*n =* 16) is represented by a unique line. The dotted lines indicate people who achieved a >log_10_ 0.5 IU/mL decline between biopsies, while unbroken lines indicate the people who had smaller HBsAg declines.

We applied the multiplex ddPCR assay to paired liver biopsies from 16 HIV/HBV-coinfected persons enrolled in the Hepatitis B Research Network Ancillary Study (16) at Johns Hopkins Hospital (Table 1). Each participant had fresh-frozen paired liver biopsies separated by a median of 3.5 years (range: 2.7–3.8 years). At biopsy 1, 12 of 16 people were on NUC therapy for more than 1 year and all were on therapy between biopsies. All but 2 people at biopsy 1 and 1 at biopsy 2 had CD4^+^ T cell counts of greater than 200 cells/mm^3^ (median 558 cells/mm^3^ and 659 cells/mm^3^ at biopsies 1 and 2, respectively). HIV RNA was undetectable in 13 of 16 individuals at each biopsy. At biopsy 2, the maximum HIV RNA was 1.7 log_10_ copies/mL. HBV DNA was detectable in 8 of 16 and 6 of 16 people at biopsy 1 and 2, respectively, and the median HBV DNA change between biopsies was –1.25 log_10_ IU/mL (IQR: –6.3 to 0.3 log_10_ IU/mL). None of the participants had HBsAg loss during treatment. The median HBsAg levels at biopsies 1 and 2 were similar (3.2 log_10_ [IQR: 3.0–3.6 log_10_] IU/mL and 2.9 log_10_ [2.8–3.3 log_10_] IU/mL, respectively; *P =* 0.3). Overall, the median HBsAg level changed minimally between biopsies by a median –0.26 log_10_ IU/mL (IQR: –0.15 to –0.48 log_10_ IU/mL decline). Notably, 4 (25%) people achieved greater than 0.5 log_10_ IU/mL HBsAg decline (range: 0.52–1.96 log_10_), whereas the median decline in the other 12 people was 0.23 log_10_ IU/mL (IQR: 0.09–0.3 log_10_ IU/mL; Figure 2). We stratified participants by the magnitude of their decrease in HBsAg quantities, comparing results of the 4 people in the highest quartile (>0.5 log IU/mL decline) to the remaining 12 individuals to test whether the magnitude of HBsAg decline during antiviral treatment could be attributed to the amount of iDNA-derived *S* mRNA.

**Figure 3:**
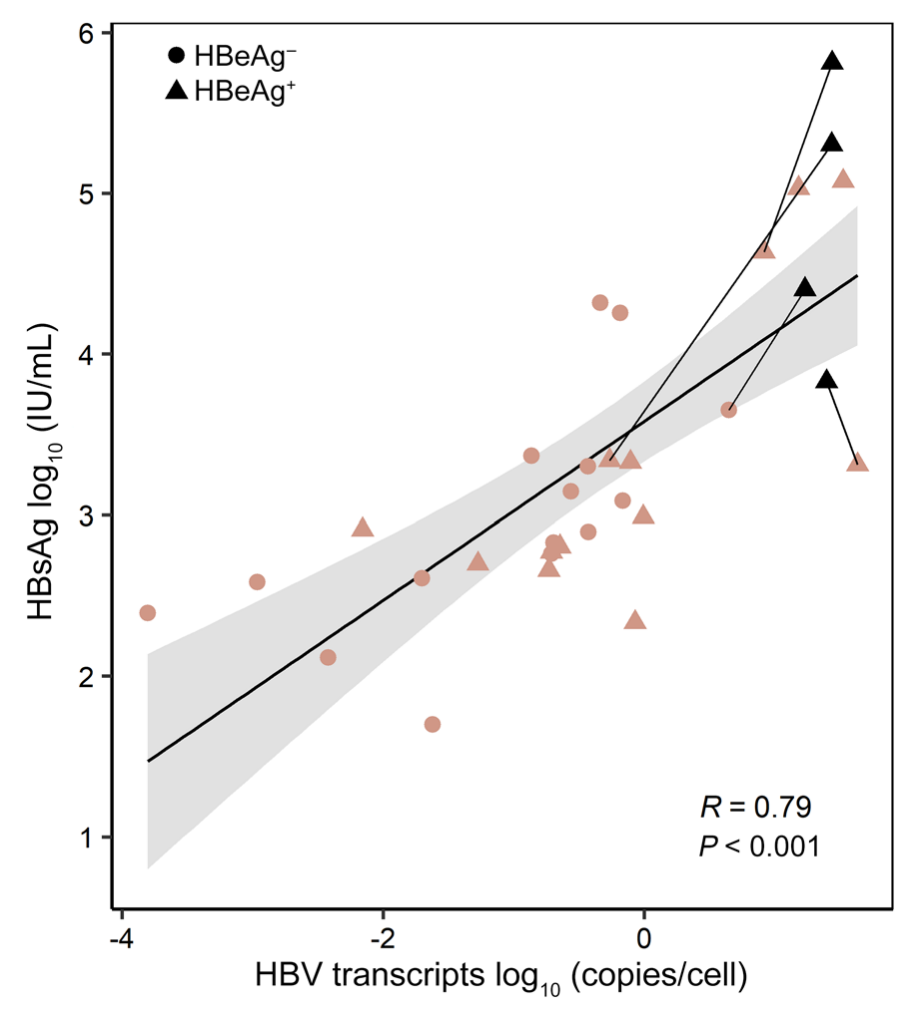
HBV transcriptional activity and serum HBsAg levels. Total HBV transcriptional activity in liver was measured by the mid-HBV assay that targets all HBV transcripts (except low-abundance 0.7 kb transcripts) and is located in the middle of the *S* ORF. Quantities were adjusted for the number of cells in each sample using ERV-3 quantities. HBV transcriptional activity in liver was correlated with contemporaneous serum HBsAg levels. Each data point represents 1 person at each biopsy (*n =* 32) and the black trendline was calculated using simple linear regression, while the shaded region depicts the 95% confidence intervals along the trendline. Black symbols depict the 4 individuals with >log_10_ 0.5 IU/mL declines in serum HBsAg at biopsy 1 with lines drawn to biopsy 2 for each person. Spearman’s correlation coefficient is shown with its *P* value and the shape of symbols (circles or triangles) corresponds to HBeAg status at the time of biopsy, as indicated in the legend.

First, we confirmed that quantifying total HBV transcription, whether from cccDNA or iDNA, was an accurate correlate of HBsAg production. We applied the assay to RNA extracted from bulk liver tissue from the 32 (16 pairs) liver biopsies. We simultaneously quantified the total number of cells in each sample using a separate qPCR assay measuring *ERV3* DNA, an endogenous retroviral sequence in every human cell (17). Because the mid-HBV assay measures all viral transcripts from cccDNA and iDNA except for the low-abundance 0.7 kb *HBX* gene product (see Methods), we used this assay to estimate total HBV transcriptional activity. Adjusting to *ERV3* quantities, HBV transcription/cell correlated closely with HBsAg levels (*r* = 0.79, *P <* 0.001; Figure 3). This result was expected, since the mid-HBV assay targets an amplicon embedded in all *S* mRNAs, irrespective of their origin. Thus, we concluded that the mid-HBV assay is an adequate surrogate for *S* mRNAs that are translated into HBsAg, and that we could use the assay multiplexed with the 3′ HBV assays to unravel the source of HBsAg production. We also considered how HBV transcription varied with hepatitis B e antigen (HBeAg) status; HBsAg levels were marginally elevated in persons who were HBeAg positive (median 3.33 log_10_ IU/mL) compared with HBeAg negative (2.90 log_10_ IU/mL; *P =* 0.069; Supplemental Figure 4D). Correspondingly, biopsies from HBeAg-positive individuals had higher levels of HBV transcription, quantified by the mid-HBV assay, compared with biopsies from HBeAg-negative individuals (*P <* 0.01; Supplemental Figure 4A).

### iDNA transcription in liver explains the response of serum HBsAg levels to NUCs.

**Figure 4:**
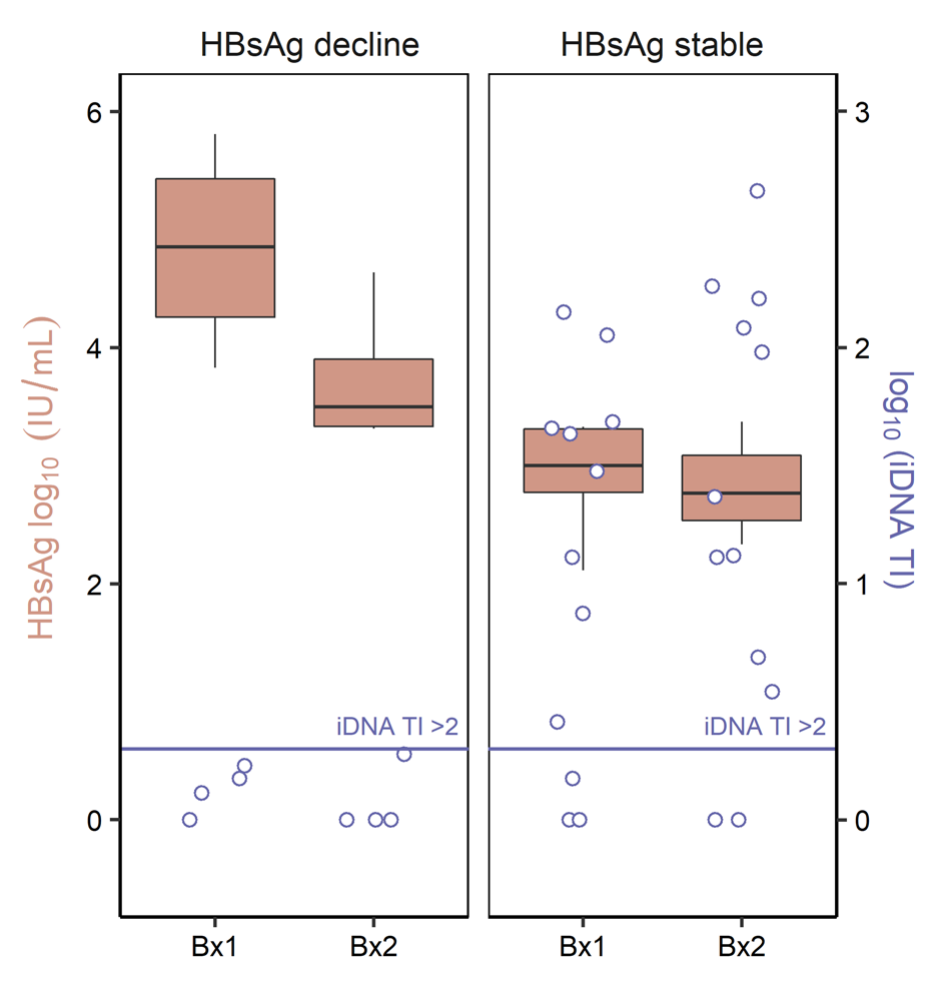
HBsAg decline during NUC treatment and iDNA-derived transcription. Box-and-whisker plots represent the summarized serum HBsAg levels at the time of each biopsy for people who achieved a >0.5 log_10_ IU/mL decline (left panel, *n =* 4) and those who had ≤0.5 log_10_ IU/mL (right panel, *n =* 12). The absolute HBsAg levels are shown on the leftward *y* axis and are visualized as the salmon-colored box-and-whiskers plots. The central horizontal line for each box-and-whisker denotes the median HBsAg amounts, while the box demarcates the interquartile range of the data. The whiskers extend to encompass ± 1.5 × IQR of the data. Data points (open purple circles) corresponding to the contemporaneous liver measurements of the iDNA transcriptional index (iDNA TI) from the same people are overlaid and quantified by the rightward *y* axis (*n =* 32). A horizontal cutoff (purple line) indicates a sample for which the majority of HBV transcripts derive from iDNA since an iDNA TI > 2 indicates that more transcripts derive from iDNA than from cccDNA. Bx1, biopsy 1; Bx2, biopsy 2.

We next tested whether differences in iDNA transcription could explain the differences in decline in serum HBsAg between the 4 persons in the highest quartile who had a greater than 0.5 log_10_ decline during NUC therapy compared with the 12 others who did not. We calculated an iDNA transcriptional index (iDNA TI, see Methods), defined as the ratio of cccDNA- and iDNA-derived transcripts (mid-HBV amplicon) to only cccDNA-derived transcripts (3′ HBV amplicon). Thus, the iDNA TI represents a relative relationship between the quantity of iDNA- to cccDNA-derived *S* transcripts, facilitating comparison of the dominant source of *S* transcripts between biopsies regardless of the absolute quantity of *S* transcripts. An iDNA TI of 1 or less is interpreted as transcription only from cccDNA, whereas an iDNA TI of greater than 2 represents HBsAg transcripts deriving predominantly from iDNA rather than from cccDNA. An iDNA TI between 1 and 2 represents contributions from iDNA and cccDNA without dominance of either. Interestingly, we found that individuals with a 0.5 log_10_ or less decline in HBsAg were transcribing predominantly from iDNA at both biopsies 1 and 2, with median iDNA transcriptional indices of 21.5 (IQR: 2.0–47.1) and 18.3 (IQR: 4.2–141.8), respectively (Figure 4). However, in people who achieved a greater than 0.5 log_10_ decline in HBsAg while on NUC therapy, viral transcription was primarily from cccDNA at biopsies 1 and 2, with median iDNA transcriptional indices of 1.4 (range: 0.9–1.7) and 0.9 (range: 0.9–1.9), respectively (Figure 4). This difference in iDNA TI was statistically significant between those who achieved a greater than 0.5 log_10_ decline and those who did not (*P <* 0.01). We confirmed that the total amount of serum HBsAg at either time point did not confound the relationship between the decline in HBsAg and the iDNA TI (Supplemental Figure 5).

### iDNA-derived transcription is a dominant source of S mRNAs during NUC therapy.

Because iDNA and cccDNA transcription can independently contribute to HBsAg, and because we previously reported that cccDNA transcription is silenced during NUCs (3, 6, 9, 14, 15), we next examined the relationship between iDNA and cccDNA transcription in the context of NUC therapy. We have previously used the 5′ HBV amplicon to quantify 3.5 kb transcripts with a particular interest in pgRNA (14, 15). As these transcripts are almost exclusively a result of cccDNA transcription, we use this as a surrogate for cccDNA transcriptional activity, but conservatively designate the pgRNA/cccDNA molecular ratio as the pgRNA transcriptional index (pgRNA TI) (see Methods).

**Figure 5:**
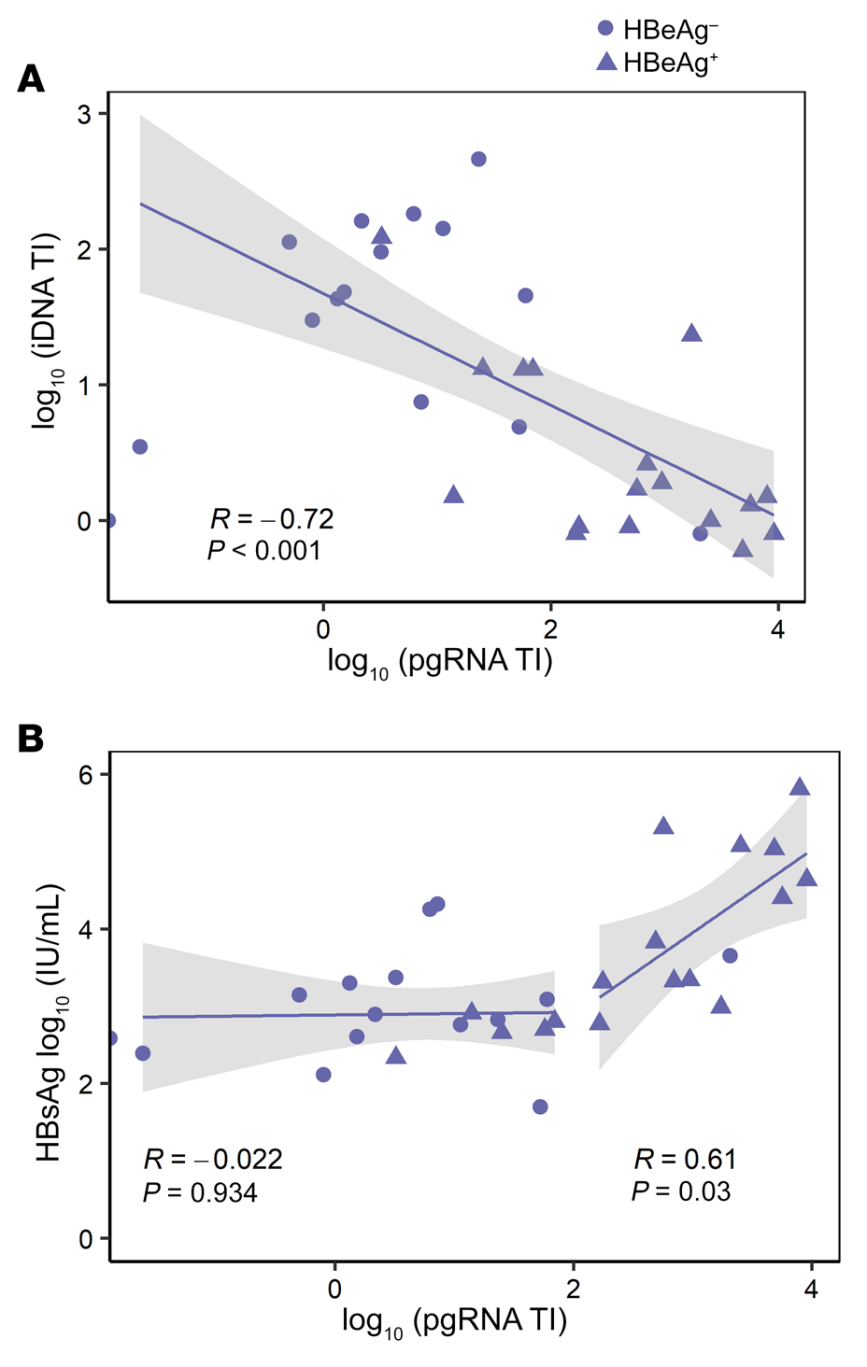
iDNA transcription and serum HBsAg in relation to pgRNA TI (surrogate for cccDNA-derived transcription). Each data point represents each person at each biopsy, with the shape of the symbol indicating HBeAg status at the time of biopsy (*n =* 32). Trendlines were calculated using a simple linear regression, with shading indicating the 95% confidence intervals around the lines. (**A**) Spearman’s correlation coefficient shows the relationship between iDNA TI and pgRNA TI, a measure of cccDNA transcription. (**B**) Serum HBsAg levels are shown correlated with the pgRNA TI in 2 components. To the right are shown points and a simple linear regression from people with ≥100 pgRNA transcripts/cccDNA molecule wherein all people had been NUC suppressed for <5 years. To the left are shown people <100 pgRNA transcripts/cccDNA molecule, the majority of whom had been NUC suppressed for ≥5 years. Spearman’s correlation coefficients and *P* values corresponding to each group are shown under each component.

As in our prior single-cell studies (14, 15), we found that NUC therapy duration was associated with reductions in cccDNA transcription (pgRNA TI) in bulk tissues (*r* = –0.51, *P <* 0.01; Supplemental Figure 6). Interestingly, while transcription from cccDNA during NUCs diminished, the relative transcription from iDNA increased; we observed that the pgRNA TI and iDNA TI were inversely correlated (*r* = –0.72, *P <* 0.001; Figure 5A). This finding suggests that cccDNA transcription contributed less to *S* mRNAs during NUC therapy, as iDNA transcription produced an increasing fraction of *S* mRNAs. This transcriptional switching parallels HBeAg status; HBeAg-negative persons were pgRNA TI^low^/iDNA TI^high^ and HBeAg-positive persons were pgRNA TI^high^/iDNA TI^low^ (iDNA TI *P <* 0.01, pgRNA TI *P <* 0.001, both comparing HBeAg-positive to HBeAg-negative people; Supplemental Figure 4, B and C).

To test whether cccDNA transcriptional silencing could be independently linked to HBsAg declines, we plotted the pgRNA TI against serum HBsAg (Figure 5B). Intriguingly, we found that decline from high to low levels of cccDNA transcription (pgRNA TI) correlated with a decline in serum HBsAg levels to a threshold pgRNA TI of approximately 100 (*r* = 0.61, *P <* 0.03). Below that threshold, serum HBsAg levels did not appear to decline any further (*r* = –0.022, *P =* 0.9).

### Single-cell viral transcriptional landscapes reveal the contributions of iDNA versus cccDNA.

**Figure 6:**
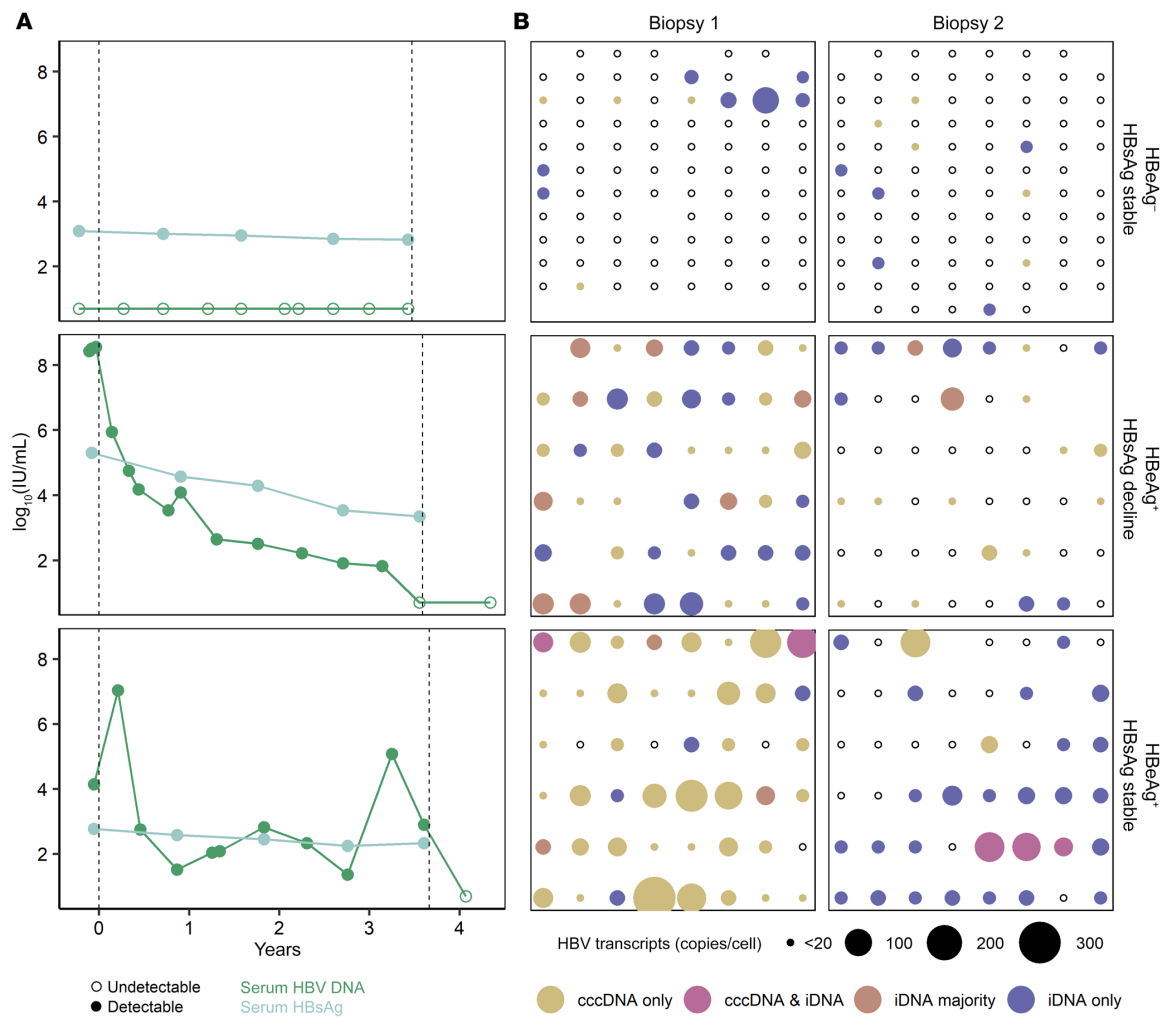
HBV serology and single-cell viral transcriptional landscapes from 3 representative people over time. (**A**) Serum HBV DNA (dark green) and serum HBsAg (slate) levels between biopsies are represented for each person who underwent single-cell examination (*n =* 3). All available time points are shown and open circles are those that were below the limit of detection for each assay. Vertical dashed lines indicate when biopsies were taken from each person. (**B**) Each data point represents a single hepatocyte after QC exclusion. Grids are arrayed in the *x*-*y* plane to reflect their position in a contiguous section of tissue. Three representative people are shown at each biopsy. The size of each data point corresponds to the quantity of HBV transcripts/cell, as measured by the mid-HBV assay, and open points indicate that no HBV transcription was detected. Colors indicate the source of viral transcripts in cells, as specified in the legend and determined by the iDNA TI. Some cells were found to have cccDNA-derived transcription but had undetectable *S* transcripts; these were colored gold and are shown as the smallest points. All cells that were deemed analyzable (because they passed QC by having sufficient total RNA) are depicted, irrespective of their infection or viral transcriptional status.

To further understand how iDNA maintains levels of HBsAg in the liver during NUC therapy, we examined individual hepatocytes from 3 individuals. We performed scLCM (14, 15) on paired biopsies from 3 of the 16 study participants who were representative of others in the cohort (Table 2): (a) an individual who was HBeAg negative and experienced minimal HBsAg decline, (b) an individual who was HBeAg positive and experienced 1.96 log_10_ IU/mL HBsAg decline, and (c) an individual who was HBeAg positive and experienced minimal HBsAg decline. We included analyses of the participants’ serum HBV DNA that were available through the cohort as a surrogate for the presence and efficacy of NUC suppression of viral replication. We studied an array of hepatocytes, spatially consecutive as we have done previously, from each biopsy for the 3 persons. We applied multiplex ddPCR using the 5′, mid-, and 3′ HBV amplicons to the RNA extractions from each cell. We developed an algorithm using the iDNA TI to classify each cell by the relative contribution of iDNA transcription to *S* mRNAs: (a) transcribing only from cccDNA (iDNA TI ≤ 1), (b) transcribing from cccDNA and iDNA (1 < iDNA TI ≤ 2), (c) transcribing predominantly from iDNA (iDNA TI > 2), and (d) transcribing only from iDNA (mid-HBV^+^/3′ HBV^–^; Figure 6B). Thus, after excluding cells with incomplete measurements (26 of 384; 6.7%), the remaining cells could be indexed for transcriptional activity and the relative contributions from cccDNA or iDNA.

We designated a cell as transcriptionally active if either the 5′, mid-, or 3′ HBV amplicons were detectable. In the 2 HBeAg-positive individuals, we observed that some cells transcribed *S* more from iDNA relative to cccDNA, whereas others transcribed more from cccDNA (Figure 6B). In contrast, in the HBeAg-negative individual, all cells with *S* mRNAs transcribed chiefly from iDNA.

In the HBeAg-negative individual with stable HBsAg levels and undetectable HBV DNA at both biopsies (Figure 6A), the proportion of transcriptionally active cells between biopsies was stable: 11 of 83 (13%) cells at biopsy 1 and 10 of 90 (11%) cells at biopsy 2. Of these transcriptionally active cells, 63% and 50% at biopsy 1 and 2, respectively, transcribed predominantly from iDNA relative to cccDNA (Figure 6B), although this difference was not statistically significant. Compared with bulk, there was a lack of enrichment of iDNA-derived transcripts between biopsies in this individual, although the single-cell work was not powered to estimate precise changes in iDNA transcription enrichment. In the HBeAg-positive individual with 1.96 log_10_ IU/mL HBsAg decline between biopsies, the proportion of transcriptionally active cells decreased between the biopsies from 45 of 45 (100%) cells at biopsy 1 to 22 of 46 (48%) cells at biopsy 2 (*P <* 0.001; Figure 6B). This decrease in cellular transcriptional activity paralleled the declines in serum HBV DNA (Figure 6A). Notably, the proportion of transcriptionally active cells that predominantly derived transcripts from iDNA was similar at biopsy 1 and 2 (56% and 45%, respectively). Further, the pgRNA TI from bulk tissue did not decline, suggesting that hepatocytes that remained infected were transcribing from cccDNA at a steady rate. However, we did observe a considerable loss of detectable cccDNA in bulk tissues (Table 2). The decline in HBsAg in this HBeAg-positive individual, therefore, is best explained by a loss of infected hepatocytes, especially those that were transcribing primarily from cccDNA, rather than a change in transcription from either iDNA or cccDNA. This is in stark contrast to the HBeAg-positive individual with stable HBsAg between biopsies (Figure 6A). While similar to the HBeAg-positive person with a significant HBsAg decline in that more cells at biopsy 1 than 2 were transcriptionally active (biopsy 1, 44 of 48 [92%] cells; biopsy 2, 29 of 46 [63%] cells; Figure 6B), the fraction of transcriptionally active cells that derived *S* mRNAs from iDNA was enriched over time: from 7 of 44 (16%) cells at biopsy 1 to 24 of 29 (83%) at biopsy 2. This finding aligns closely with what we found in bulk tissue, where the iDNA TI increased from 0.8 to 121.6 (Table 2). Thus, we hypothesize that in this individual, despite loss of infected hepatocytes, there was enrichment in the proportion of cells transcribing from iDNA that ultimately led to stable levels of serum HBsAg. Taken together, our results show that while NUC-associated cccDNA transcriptional silencing is occurring, HBsAg levels appeared to be governed by whether and to what extent iDNA transcription occurred.

## Discussion

We developed a multiplex ddPCR assay to distinguish cccDNA- from iDNA-derived *S* transcripts and applied it to both bulk liver tissue and individual hepatocytes in paired liver biopsies from 16 individuals during 2 to 4 years of NUC therapy as a part of highly active antiretroviral therapy. We found that individuals who are HBeAg positive have greater relative *S* transcription from cccDNA, whereas those who are HBeAg negative transcribe *S* predominantly from iDNA. Interestingly, for individuals in whom iDNA was the primary source of *S* transcripts at baseline, NUC treatment did not substantially reduce the levels of HBsAg in blood. In contrast, NUC treatment did reduce the levels of HBsAg in blood in individuals in whom cccDNA was the predominant source at baseline and we attribute this to cccDNA transcriptional silencing and clearance of infected cells. Our single-cell data largely corroborate these findings but also allow a more granular view of how relative changes in iDNA versus cccDNA transcription within single hepatocytes relate to changes in total HBV transcription. Overall, our results highlight the importance of iDNA transcription in some individuals to sustain elevated HBsAg levels despite NUC treatment of CHB; in those individuals, the relative amount of iDNA- to cccDNA-derived transcription appeared to enrich over time. Thus, transcriptionally active iDNA contributes to circulating HBsAg during NUCs and is therefore a barrier to functional cure.

We clearly demonstrate that HBeAg-positive individuals transcribe *S* mostly from cccDNA, whereas HBeAg-negative individuals transcribe *S* predominantly from iDNA. This difference in the origin of *S* transcripts was highlighted by Wooddell et al. when treating HBeAg-positive and HBeAg-negative chimpanzees, after NUC lead-in, with an siRNA targeting the shared 3′ end of cccDNA- but not iDNA-derived viral transcripts (8). HBeAg-positive chimpanzees had significant HBsAg declines and robust cccDNA transcription in contrast to HBeAg-negative chimpanzees with stable HBsAg levels and predominantly iDNA transcription. After adjusting the siRNA target to silence both cccDNA- and iDNA-derived transcripts, HBsAg levels declined universally, highlighting the contribution of iDNA-derived *S* mRNAs to HBsAg production. Long-read sequencing of RNA from 2 chimpanzees in this study showed that 77% of transcripts derived from iDNA in the HBeAg-negative chimpanzee, compared with 10% from iDNA in the HBeAg-positive chimpanzee. Two other groups have reported a similar phenomenon in the context of hepatocellular carcinoma (HCC), as liver from people with HCC had 80% to 90% of viral transcripts deriving from iDNA rather than cccDNA, though HBeAg status was not available for these studies (18, 19). Although HBsAg levels in our 16 study participants were higher in HBeAg-positive individuals than in HBeAg-negative individuals, the difference was only borderline significant, highlighting that iDNA transcription in HBeAg-negative individuals can lead to high levels of HBsAg. There is also heterogeneity between individuals in the relative transcription from iDNA. This may be determined during the initial infection since increased initial viremia is associated with increased viral integration and prolonged HBsAg levels (10, 20). It is intriguing to consider that earlier treatment may limit integration events and allow for more effective control of HBsAg production with NUCs.

We and others have reported that NUC therapy is associated with cccDNA transcriptional silencing, which can also be inferred from the decrease in HBV RNA in serum as well as pgRNA in the liver (2, 6, 9, 10, 14, 15). By comparing quantities of pgRNA to cccDNA in people taking NUCs who had decreases in serum HBV DNA, here we confirmed our earlier findings that NUCs are associated with transcriptional silencing of cccDNA (14, 15), rather than the alternative hypothesis that HBV transcription is reduced during NUCs strictly because of an overall reduction in the number of infected cells. cccDNA transcriptional silencing during NUCs is likely to be transient, since treatment interruption is frequently associated with virologic rebound. After HBV DNA is no longer detectable in serum and HBV RNA has declined, substantial HBsAg quantities remain in the majority of NUC-treated persons, even if diminished (2, 3, 8, 13, 21). Our data offer a potential explanation for this previously unexplained observation in that the individuals who transcribed *S* mostly from cccDNA had significant HBsAg declines coupled with decreases in cccDNA transcription on NUC therapy, whereas those who transcribed mostly from iDNA did not have substantial declines in HBsAg levels. These findings suggest that NUC therapy may modulate HBsAg levels in cccDNA transcriptionally dominant individuals but not in iDNA transcriptionally dominant individuals. A recent study by van Buuren et al. showed a similar phenomenon in individuals treated with NUC plus PEG-IFN-α; iDNA-derived transcription was enriched when HBsAg levels remained stable, although the group was unable to determine whether this was an effect of PEG-IFN-α or NUCs since all participants received PEG-IFN-α (12). We now demonstrate enrichment of iDNA transcription relative to cccDNA transcription with NUCs, especially when cccDNA-derived transcription is progressively diminished. These observations may impact treatment strategies for people with CHB, as currently NUC therapy is required lifelong to suppress HBV DNA and HBV RNA levels in serum. Our findings establish that the subset of people with HBsAg deriving mainly from iDNA may never achieve further declines in HBsAg from prolonged NUC therapy alone since NUCs are not known to inhibit transcription from iDNA, although they likely interrupt new integration events. We predict that the subset of individuals with primarily iDNA-derived HBsAg will require novel therapies targeting iDNA-derived *S* mRNAs or the cells that produce them.

Our single-cell analysis highlights that HBV transcription is not static in the liver even though it may appear so when strictly looking at HBsAg levels. In the HBeAg-positive individual with stable HBsAg levels between biopsies (Figure 6), the production of *S* transcripts switched from cccDNA to iDNA dominant. Whether this is due to prolonged NUC treatment or changes in host factors deserves further exploration. The single-cell work also supports the findings that HBsAg loss is mainly due to diminished cccDNA transcription, possible cccDNA degradation, or loss of cells containing cccDNA, as evidenced by the HBeAg-positive individual who had a decline in HBsAg coupled with a loss of detectable cccDNA in bulk. A conjecture that arises from the single-cell work is whether enrichment of iDNA transcriptionally active cells is indicative of clonal proliferation, if indeed NUCs completely suppress viral replication, which itself remains an open question. Each of the individuals selected for single-cell work had different HBsAg trajectories or a different HBeAg status; thus, further single-cell work is needed to determine whether there are other patterns of changes in individual hepatocytes over time with NUC therapy.

Current methods to determine the origin of a viral transcript as cccDNA or iDNA are cumbersome, expensive, and have a high degree of quantification error, as they rely on Alu-PCR, RNA-seq, or sequential PCR and subtraction strategies (10, 12, 20). Our assay is not only broadly applicable for multiple genotypes, but also multiplexed so that quantification of total HBV transcripts and cccDNA-derived transcripts happens within the same reaction, thus abrogating the need for subtraction strategies. Moreover, our assay directly quantifies RNAs that correlate closely with absolute HBsAg levels (Figure 3), without the need for intermediate steps such as library generation. The multiplex ddPCR format is therefore readily applicable and relatively inexpensive, in contrast to other platforms (3, 21, 22).

Although our tool offers insight into clinical observations, there are several limitations to this study. Our assay may not be optimized for genotypes B, G, and H, which were not represented in our cohort. We anticipate that the key findings in those genotypes would be similar, although a pangenotypic assay would be important to study global HBV. While we performed an intensive study of HBV transcription that included single-cell analyses, we only studied 16 people, all of whom were male, coinfected with HIV, and the majority were African American. In the future, we will expand our work to larger and more diverse cohorts, including persons who are treatment naive and persons who are on stable antivirals, persons with HBV monoinfection, and females. We anticipate that while our findings may be consistent irrespective of which NUC is used, it will be important to apply our assay to tissues obtained from people taking novel antivirals with different mechanisms of action. We separately note that while it will be important to test how HIV affects HBV integration and transcription, HIV is unlikely to affect the overall implications of our study since the phenomenon of persistent HBsAg on NUCs is consistent between people with and without HIV infection. In addition, even if the absolute numbers of both iDNA- and cccDNA-derived *S* are higher in people with HIV, our conclusions are based on the ratio of iDNA-derived/cccDNA-derived *S* transcripts and therefore account for higher levels of replication that can be seen in people with HIV. Another limitation is that we intentionally quantified iDNA-derived transcripts rather than iDNA specifically since our principal aim was to quantitatively link the source of *S* mRNA transcription and HBsAg levels.

We report a multiplex ddPCR assay that we applied to bulk liver tissue and single hepatocytes from persons with CHB, providing evidence that iDNA transcription of *S* is responsible for the persistent HBsAg in blood in the majority of individuals receiving long-term NUCs. Our findings have 2 major implications for functional HBV cure. First, therapeutically achieving a functional cure, as it is currently defined, will require durable transcriptional silencing of hepatocytes transcribing *S* primarily from cccDNA with NUCs or other novel agents and additionally targeting cells transcribing *S* primarily from iDNA, either by eliminating them or suppressing their production of *S*. The second implication is whether the definition of functional cure requiring complete loss of HBsAg needs to be reconsidered since it may be difficult to address all hepatocytes with iDNA.

## Methods

### Study participants.

Participants were enrolled in the HIV-HBV Cohort Ancillary Study of the Hepatitis B Research Network at Johns Hopkins Hospital. Liver biopsies were obtained from each individual at 2 time points separated by 2 to 4 years. Each biopsy was placed immediately in OCT media, flash frozen at bedside, and placed in liquid nitrogen until use. Liver tissue was sectioned at 10 μm and mounted on PEN membrane slides (Supplemental Table 1) for scLCM of spatially contiguous cells in a grid to develop viral landscape maps, as previously described (14, 15, 17). For the HBeAg-negative individual, we studied twice as many cells given the relative absence of infection. Individual cells were excluded from analysis if the total RNA in a cell was not within the normal range of detection defined by greater than 1 standard deviation from 8 tissue-negative PEN membrane cuts, as assayed by *7SL*, to avoid analysis of cell fragments (Supplemental Table 1). For bulk analysis, multiple slides from each biopsy were scraped into DNA/RNA lysis buffer (Supplemental Table 1) for extraction. Hep3B cells were obtained from the American Type Culture Collection (HB-8064).

### DNA/RNA extraction, treatment, and ddPCR.

DNA and RNA were extracted (Supplemental Table 1) from the same sample simultaneously to limit sampling error for both bulk slide scrapes and scLCM samples, as previously described (14, 15, 17). To quantify cccDNA, half of each DNA sample was treated with exonuclease I/III (Supplemental Table 1), as previously described, digesting all linear and partly circular DNA (14, 15). Exonuclease I/III–treated DNA was assayed by ddPCR (Supplemental Table 1) using the mid-HBV assay (Supplemental Table 2) to detect cccDNA, as previously described (14, 15). DNase-treated (Supplemental Table 1) RNA samples were converted to cDNA (Supplemental Table 1) and any remaining RNA was digested with RNase H as the last step of reverse transcription. cDNA was analyzed by ddPCR using the 5′, mid-, and 3′ HBV assays (Supplemental Table 2). All ddPCR runs used the following cycling parameters: 1 cycle of 94°C for 10 minutes, 40 cycles of 94°C for 30 seconds and 57°C for 1 minute, 1 cycle of 98°C for 10 minutes, 1 cycle of 12°C for 10 minutes, and 1 continuous cycle at 4°C until reading. Plates were read on the QX200 Droplet Reader (Bio-Rad) for ddPCR.

### Transcriptional indices.

During reverse transcription, the pgRNA tail contains a direct repeat 1 (DR1) that acts as a primer forming a DR1-DR2 hybrid for the viral polymerase to complete rcDNA. Approximately 10% of the time, DR1 acts as an RNA primer for another DR1 in a distinct location in the DNA to make double-stranded linear DNA (dslDNA) (for comprehensive reviews on iDNA refer to refs. 23, 24). This truncation is further exacerbated when dslDNA reenters the nucleus and is digested at its ends during nonhomologous end joining at double-stranded DNA breaks in host chromosomes, resulting in the viral genome’s integration. As a result, iDNA is lacking the 5′ and 3′ ends of the HBV genome, losing promoters for all 3.5 kb transcripts, including pgRNA (Figure 1). As the promotors for 2.4, 2.1, and 0.7 kb transcripts are still present and active, these transcripts can be actively made from iDNA, though they lack the HBV PAS and instead rely on host PAS, forming virus-human chimeric mRNAs. By multiplexing the mid-HBV and 3′ HBV assays using ddPCR, we calculated an iDNA TI by dividing the number of transcripts originating from both iDNA and cccDNA (mid-HBV amplicon positive) to those originating solely from cccDNA (mid-HBV and 3′ HBV amplicon positive). We assumed, as has been previously reported, that the contribution of the 0.7 kb transcript (*HBX*) to calculations of total HBV transcription in human tissue would be negligible (25–27). Interpretations of the iDNA TI are based on comparable efficiencies of the mid-HBV and 3′ HBV assays so that a single template mRNA derived from cccDNA would be detected equivalently by these 2 assays (Supplemental Figure 3). Therefore, (a) if the iDNA TI is 1 or less, transcripts originate from cccDNA solely, (b) if greater than 1 and less than or equal to 2, transcripts originate from both cccDNA and iDNA, (c) if greater than 2, the majority of transcripts originate from iDNA. There is also the special case of zero quantifiable cccDNA-derived transcription in the presence of iDNA-derived transcription and detectable cccDNA, which we interpret to mean that cccDNA transcription is fully suppressed. The pgRNA TI was calculated, as previously published, by dividing the number of pgRNA transcripts, measured by the 5′ HBV amplicon in RNA, by the number of cccDNA molecules, quantified by the mid-HBV assay in exonuclease I/III–treated DNA (14, 15).

### Statistics.

All Spearman’s correlation coefficient analyses and linear models were performed in R using “ggpubr” (28). Significance displayed as *P* values was calculated using Wilcoxon’s rank sum and signed tests except the percentage difference in transcriptionally active cells calculated by a significance test for a difference in 2 proportions. A *P* value of less than 0.05 was considered significant. Primer/probe fidelity (Supplemental Table 3) analysis was performed using BLAST on Linux Ubuntu and then R, which demonstrates the ability to capture all genotypes except for F, G, and H (29, 30). A tutorial for beginners on aligning oligos to HBV genotypes can be found on GitHub “HBV_primer_fidelity.”

### Study approval.

The use of tissues from humans for the present study was reviewed and approved by the Office of Human Subjects Research Institutional Review Board, IRB-3, Baltimore, Maryland, USA. All participants gave written informed consent for use of their tissues for research purposes through the Hepatitis B Research Network.

## Author contributions

TG, HSH, AB, and CLT conceptualized the study. TG, HSH, AB, and CLT developed the methodology. TG wrote the software to assess primer fidelity. TG, MT, and HSH conducted formal analyses. TG and MT carried out the investigation. TG, JQ, MSS, and RKS provided resources. Writing (original draft or review and editing): TG, HSH, MT, JQ, MSS, RKS, AB, and CLT wrote the original manuscript draft or reviewed and edited it. TG generated figures. MSS, RKS, AB, and CLT acquired funding.

## Supplementary Material

Supplemental data

## Figures and Tables

**Table 2:**
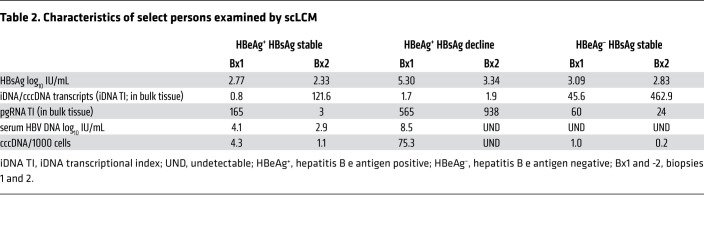
Characteristics of select persons examined by scLCM

**Table 1:**
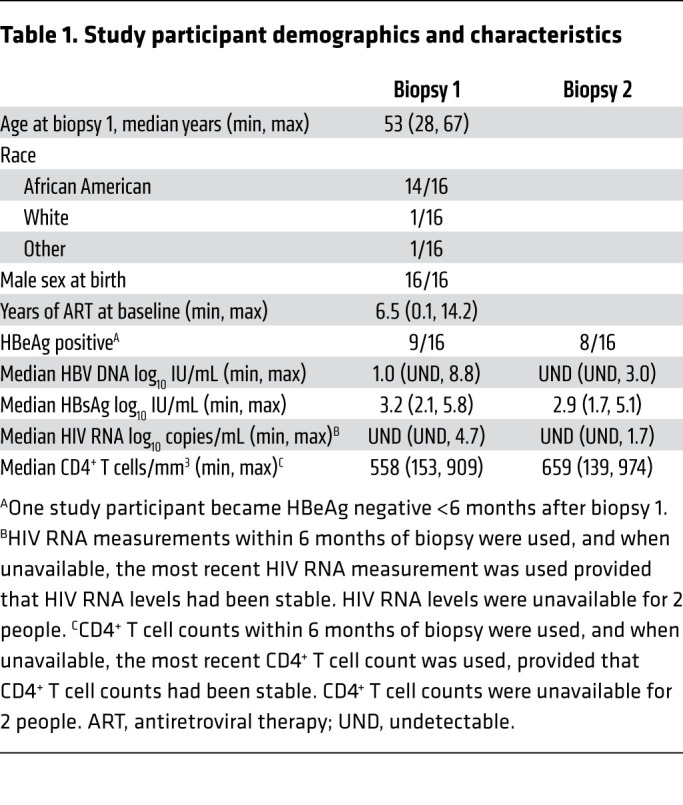
Study participant demographics and characteristics

## Version 1 07/07/2022

In-Press Preview

## Version 2 09/15/2022

Electronic publication
