# Supplementary material for: Integrated hepatitis B virus DNA maintains surface antigen production during antiviral treatment
Source: J Clin Invest. 2022 Sep 15;132(18):e161818. doi: 10.1172/JCI161818 (PMC9473722; doi:10.1172/JCI161818)
Supplement: Supplemental data [file jci-132-161818-s042.pdf]

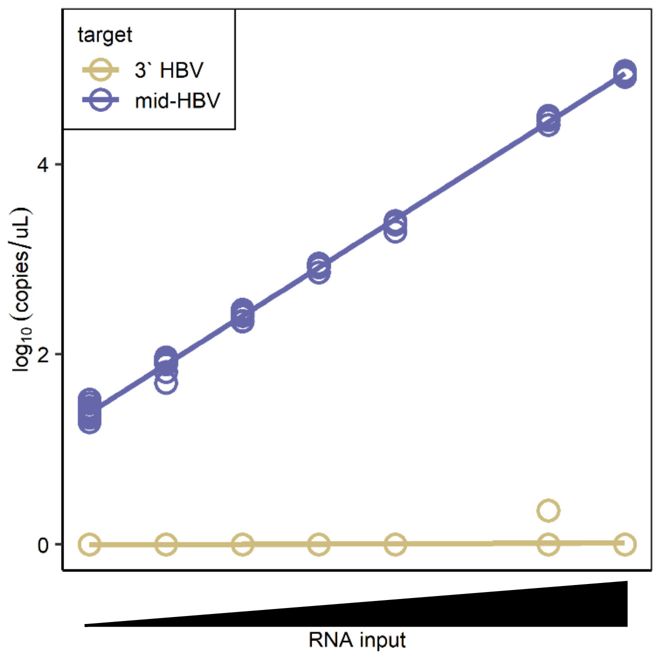

Figure S1. Assay specificity for exclusively iDNA-derived mRNAs. A Hep3B cell pellet was added to HBV-negative liver tissue that was then serially diluted prior to extraction. Replicates (n=8 for each dilution), represented by open circles, were run on ddPCR across a range of dilutions with the mid- and 3` HBV amplicons in multiplex. The lower limit of detection (LLOD) for each assay is <10 copies/μL. There was no off-target detection of HBV mRNA by the 3’ HBV assay.

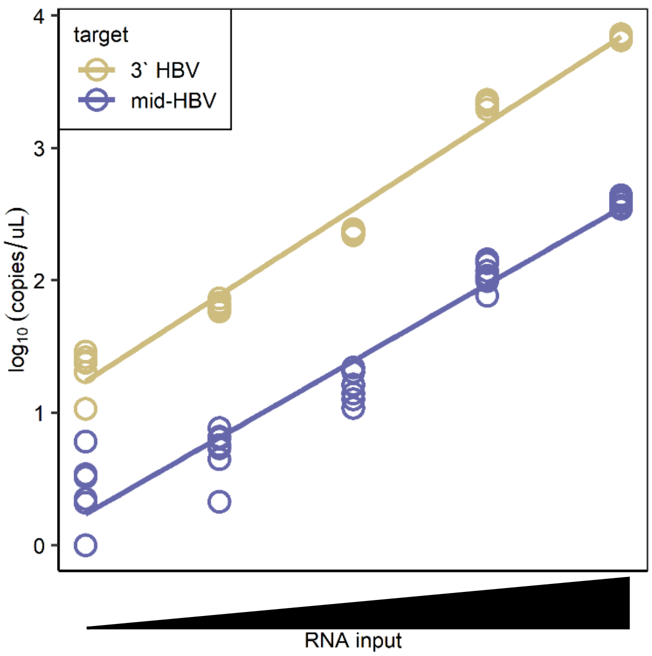

Figure S2. Assay sensitivity for cccDNA-derived mRNAs. Liver homogenates from an HBV-infected C57BL/6 mouse transfected with an HBV expression plasmid, which represents cccDNA and its resulting mRNAs, were serially diluted prior to extraction and cDNA synthesis. Replicates (n=8 for each dilution), represented by open circles, were run on ddPCR across a range of dilutions with the mid- and 3` HBV amplicons in multiplex. The lower limit of detection (LLOD) for each assay is <10 copies/μL.

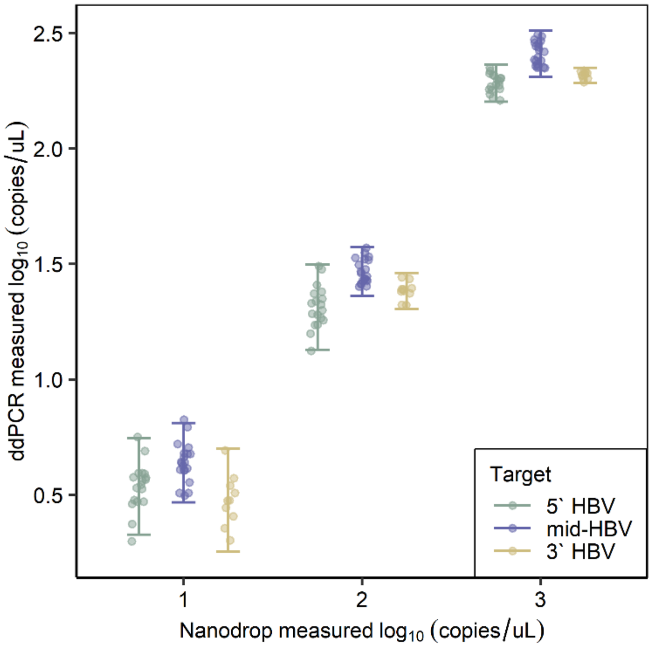

Figure S3. Assay efficiencies when measuring synthetic DNA input. Lyophilized, synthetic gBlock DNA from the manufacturer was hydrated, quantified by Nanodrop, and diluted to within the dynamic range of our assays. Dilutions where run on ddPCR with a minimum of 10 replicates for each assay to assess PCR efficiencies. Each replicate is represented as a unique point and error bars mark the spread of two standard deviations from the mean of replicates. Comparable efficiencies were shown for each assay.

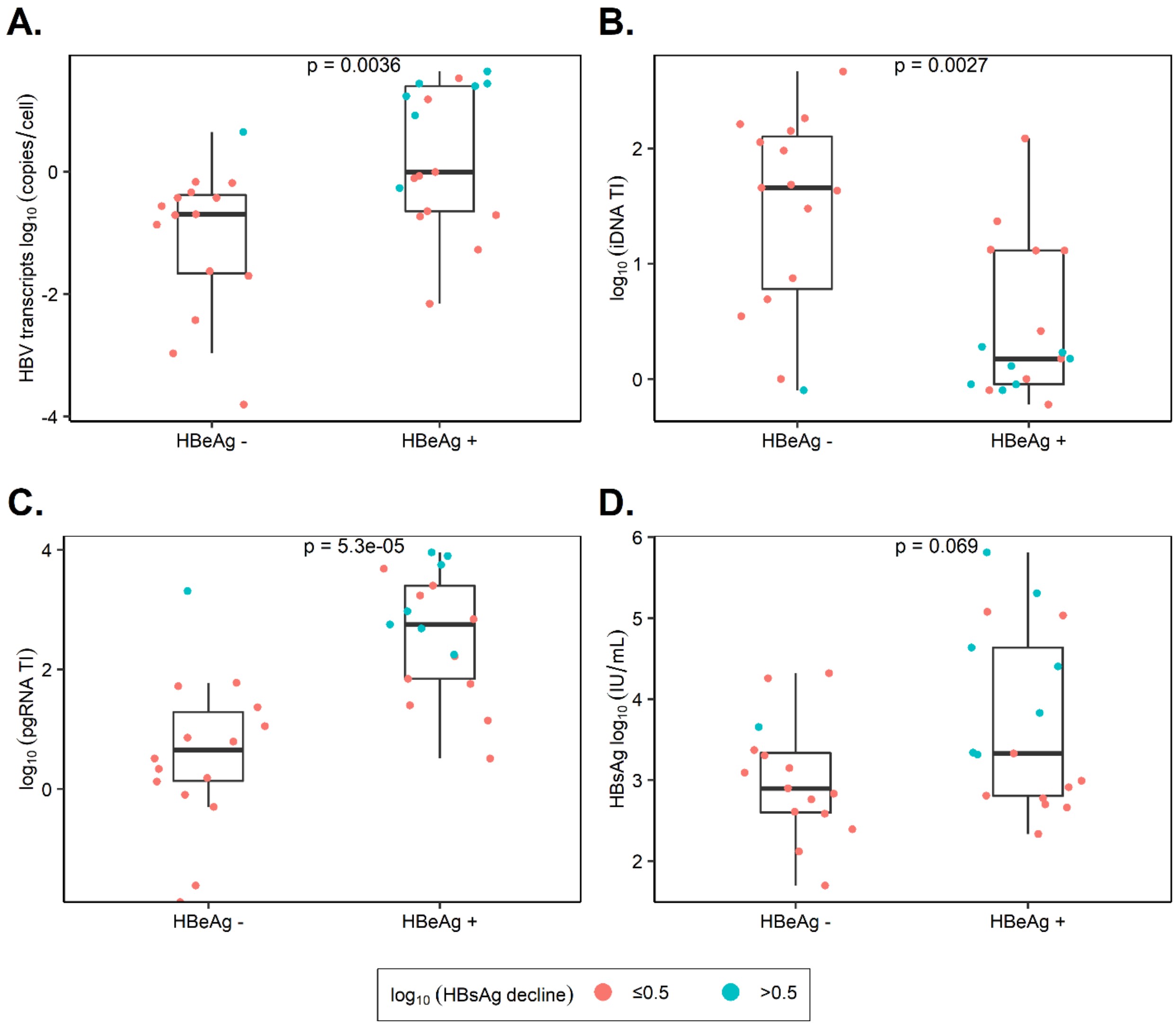

Figure S4. Study participants by HBeAg status. HBV transcription and HBsAg production was quantified and stratified by HbeAg status. For each panel, each point represents one person at each biopsy and point color corresponds to HBsAg declines within people between biopsies (n=32). One study participant was HbeAg-positive at biopsy 1 and seroconverted to HbeAg-negative by biopsy 2. Wilcoxon rank sum and signed tests were used to calculate p-values stratified by HbeAg status. A) Total HBV transcriptional activity/cell in bulk liver (quantified using the mid-HBV assay). B) iDNA TI in bulk liver. C) pgRNA TI in bulk liver. D) Serum HBsAg.

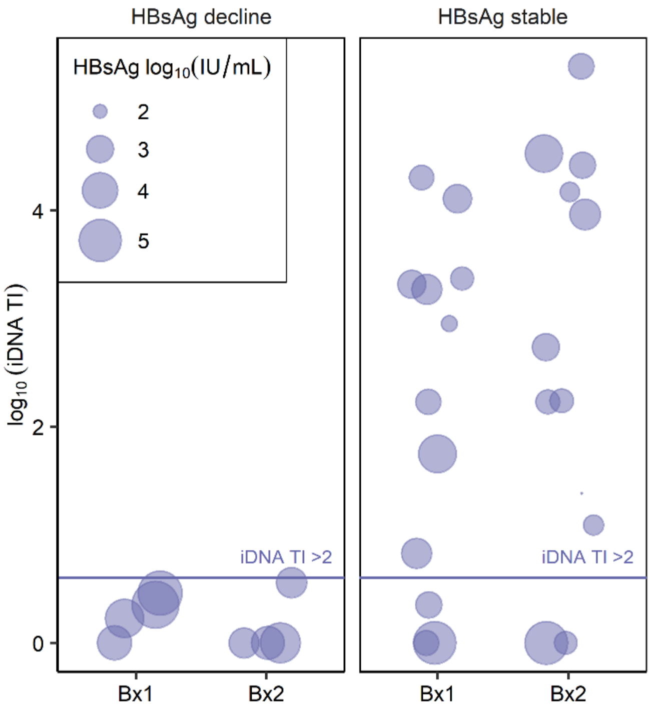

Figure S5. Relationship between absolute serum HBsAg levels and iDNA-derived S transcription. Points correspond to the leftward y-axis that displays the iDNA TI for each person at each biopsy (n=32). The sizes of points are scaled to correspond to the absolute amount of serum HBsAg. The left panel shows people who had > 0.5 $\log_{10}$ IU/mL declines in HBsAg and the right panel shows people who had ≤0.5 $\log_{10}$ IU/mL declines.

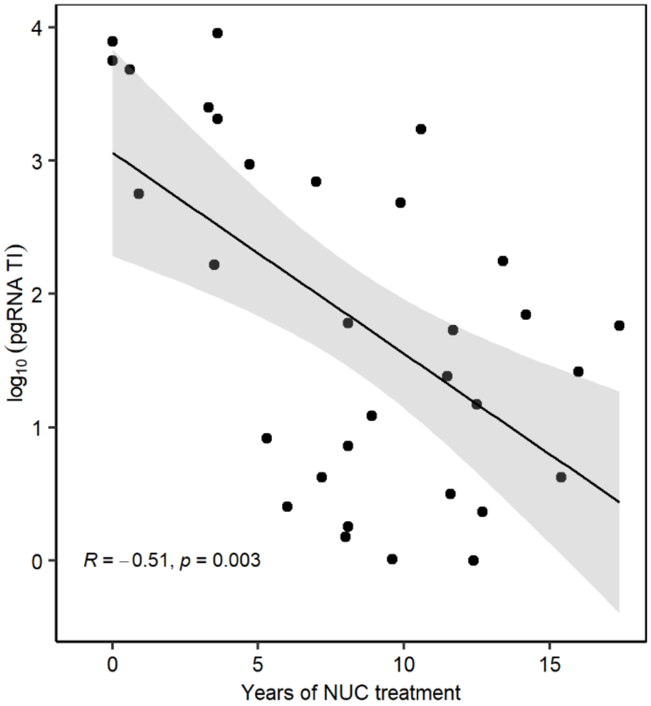

Figure S6 cccDNA transcriptional activity (pgRNA TI) in liver biopsies compared with duration of NUC therapy. cccDNA transcription was measured using the 5` HBV assay and treatment time was determined by medical record review prior to enrollment. Each point represents each person at each biopsy (n=32). Spearman’s correlation coefficient shows the inverse relationship between treatment time and cccDNA transcription.

Table S1. Reagents and samples.

Purpose	Kit/Reagent Name	Cat. #	Company
Slide scrape lysis	DNA/RNA Lysis Buffer	D7001-1-50	Zymo Research; Irvine, CA
DNA/RNA Extraction	ZR-Duet DNA/RNA Miniprep	D7001	Zymo Research; Irvine, CA
scLCM	PEN membrane (2μM) coated glass slides	11505158	Leica Microsystems; Wetzlar, Germany
DNase I	DNase I Set	E1010	Zymo Research; Irvine, CA
cDNA synthesis	SuperScript IV First-Strand Synthesis System	18091050	Thermo Fisher; Waltham, MA
ddPCR	ddPCR Supermix for Probes (No dUTP)	1863025	Bio-Rad; Hercules, CA
Primer efficiency comparisons	gBlock Gene Fragments		Integrated DNA Technologies
Cell quantification	*ERV3* qPCR primer/probe mix	Hs.PT.58.25936050	Integrated DNA Technologies
scLCM quality control	*7SL* qPCR primer/probe mix	Hs.PT.58.24302063.g	Integrated DNA Technologies

Table S2. Primers and probes for ddPCR

**Target**	**Sequence**	**position from EcoRI cut site**
**5` HBV Forward**	**CACCTCTGCCTAATCATC**	**1826-1843**
**5` HBV Reverse**	**GGAAAGAAGTCAGAAGGCAA**	**1853-1877**
**5` HBV Probe**	**TGTCCYACTGTTCAAGCCTCCAAGC**	**1955-1974**
**Middle HBV Forward**	**CTCGTGGTGGACTTCTCTC**	**253-271**
**Middle HBV Reverse**	**CAGCAGGATGAAGAGGAA**	**320-344**
**Middle HBV Probe**	**CCAACCTCCAATCACTCACCAACCT**	**401-418**
**3` HBV Forward**	**TAGGAGGCTGTAGGCATAA**	**1774-1792**
**3` HBV Reverse**	**CACAGCTTGGAGGCTTGAA**	**1815-1844**
**3` HBV Probe**	**TGCAACTTTTTCACCTCTGCCTAATCATCT**	**1963-1881**

Table S3. The percentage of full-length HBV sequences of all genotypes that are addressed by each primer and probe set

Genotype (n sequences)	5` HBV (%)			Mid-HBV (%)			3` HBV (%)		
	F	P	R	F	P	R	F	P	R
A (508)	99	99	95	99	100	100	99	99	100
B (1000)	99	99	94	98	3	89	93	96	100
C (1543)	99	97	97	99	96	100	84	99	100
D (727)	98	98	91	100	99	99	97	95	100
E (230)	100	97	96	100	98	100	98	98	99
F (170)	99	100	2	99	100	100	99	96	100
G (20)	100	95	0	100	0	100	100	60	95
H (17)	94	100	0	100	0	100	100	82	100
I (29)	100	97	93	100	97	100	100	100	100

All alignments allow for up to one mismatch. F – forward primer; P – probe; R – reverse primer
